# Supplementary material for: Psychosocial Interventions to Improve Psychological, Social and Physical Wellbeing in Family Members Affected by an Adult Relative’s Substance Use: A Systematic Search and Review of the Evidence
Source: Int J Environ Res Public Health. 2021 Feb 12;18(4):1793. doi: 10.3390/ijerph18041793 (PMC7918716; doi:10.3390/ijerph18041793)
Supplement: Supplementary file 1 [file ijerph-18-01793-s001.pdf]

1. substance-related disorders/ or alcohol-related disorders/ or amphetamine-related disorders/ or cocaine-related disorders/ or drug overdose/ or inhalant abuse/ or marijuana abuse/ or opioid-related disorders/ or phencyclidine abuse/ or psychoses, substance-induced/ or substance abuse, intravenous/ or substance withdrawal syndrome/ or alcohol withdrawal delirium/ or alcohol withdrawal seizures/
2. ((stimulant* or polydrug* or drug* or substance) adj6 (abus* or dependen* or addict* or disorder* or intoxicat* or misuse*)).ab,ti.
3. exp alcohol drinking/
4. (alcohol adj3 (dependen* or drink* or intoxicat* or abus* or misus* or risk* or consum* or excess* or reduc* or intervention*)).ab,ti.
5. (drink* adj3 (excess or heavy or heavily or harm or harmful or hazard* or risky or binge or harmful or problem*)).ab,ti.
6. (addict* or abstain* or abstinen*).ab,ti.
7. (heroin or methadone or temegesic or subutex or opiate* or crack cocaine or cocaine or ecstasy or methamphetamine* or crystal meth or amphetamine* or cannabis or marijuana or marijuana or lsd or magic mushrooms or mephedrone or khat or cathinone or ketamine or gammahydroxybutrate or ghb or amyl nitrate).ab,ti.
8. 1 or 2 or 3 or 4 or 5 or 6 or 7
9. family/ or caregiver/ or parent-child relations/ or father-child relations/ or mother-child relations/ or parenting/ or paternal behavior/ or paternal deprivation/
10. (famil* or significant other or affected other* or carergiver or carer or spouse or husband or wife or wives or partner* or parent* or father* or mother* or sibling* or brother* or sister* or child* or son* or daughter* or grandparent* or grandmother* or grandfather* or relative*).ab,ti
11. 9 or 10
12. psychotherapy/ or exp behavior therapy/ or exp cognitive therapy/ or exp relaxation therapy/ or gestalt therapy/ or narrative therapy/ or nondirective therapy/
13. play therapy/ or exp psychoanalytic therapy/ or exp psychotherapeutic processes/ or psychotherapy, brief/ or psychotherapy, multiple/ or psychotherapy, psychodynamic/
14. psychotherapy, rational-emotive/ or reality therapy/
15. socioenvironmental therapy/
16. counseling/ or exp directive counseling/
17. (motivat* adj5 (interview* or therap* or consult* or intervention* or enhance*)).ab,ti.
18. (brief adj3 intervention*).ab,ti.
19. (cognit* adj2 (train* or behavior* or therap* or technique* or skill*)).ab,ti.
20. ((psychodynamic or psychosocial) adj2 (therap$ or treatment$ or intervention$ or program$)).ab,ti.
21. (psychotherap* or counsel* or residential rehabilitation).ab,ti.
22. ((relaxation or imagery) adj2 (therap$ or technique$)).ab,ti.
23. (family adj2 therap*).ab,ti.
24. (case adj2 management).ab,ti.
25. ((coping skill* or cbst or self control or assertive*) adj2 (training or therap*)).ab,ti.
26. 12 or 13 or 14 or 15 or 16 or 17 or 18 or 19 or 20 or 21 or 22 or 23 or 24 or 25
27. (randomized controlled trial or controlled clinical trial).pt.
28. (randomized or placebo).ab.
29. clinical trials as topic.sh.
30. randomly.ab.
31. trial.ti.

32. 27 or 28 or 29 or 30 or 31
33. exp animals/ not humans.sh.
34. 32 not 33
35. 8 and 11 and 34
